# The willingness to perform first aid among high school students and associated factors in Hue, Vietnam

**DOI:** 10.1371/journal.pone.0271567

**Published:** 2022-07-27

**Authors:** Le Duc Huy, Pham Thanh Tung, Le Nguyen Quynh Nhu, Nguyen Tuan Linh, Dinh Thanh Tra, Nguyen Vu Phuong Thao, Tran Xuan Tien, Hoang Huu Hai, Vo Van Khoa, Nguyen Thi Anh Phuong, Hoang Bao Long, Bui Phuong Linh

**Affiliations:** 1 Health Personnel Training Institute, University of Medicine and Pharmacy, Hue University, Hue, Vietnam; 2 School of Health Care Administration, College of Management, Taipei Medical University, Taipei, Taiwan; 3 Department of Epidemiology, Harvard T.H. Chan School of Public Health, Boston, MA, United States of America; 4 Department of Physiology, Hanoi Medical University, Hanoi, Vietnam; 5 Research Advancement Consortium in Health, Hanoi, Vietnam; 6 Daklak Center for Diseases Control and Prevention, Daklak, Vietnam; 7 University of Medicine and Pharmacy, Hue University, Hue, Vietnam; 8 Faculty of Public Health, Department of Epidemiology and Statistics, Danang University of Medical Technology and Pharmacy, Da Nang, Vietnam; 9 Department of Obstetrics and Gynecology, University of Medicine and Pharmacy, Hue University, Hue, Vietnam; 10 Faculty of Nursing, University of Medicine and Pharmacy, Hue University, Hue, Vietnam; 11 Faculty of International Education, University of Medicine and Pharmacy, Hue University, Hue, Vietnam; 12 Office of Science-Technology and International Relations, University of Medicine and Pharmacy, Hue University, Hue, Vietnam; 13 Institute of Gastroenterology and Hepatology, Hanoi, Vietnam; 14 Department of Nutrition, Harvard T.H. Chan School of Public Health, Boston, MA, United States of America; Curtin University, AUSTRALIA

## Abstract

**Background:**

Adolescents who are willing to perform first aid can help prevent injuries and ultimately death among themselves and others involved in accidents or injuries. This study aims to estimate the prevalence of students’ willingness to perform first aid procedures and additionally examine associated factors among high school students in Hue, Vietnam.

**Methods:**

A cross-sectional study utilizing multi-stage stratified random sampling was conducted between April to July 2020 by investigating 798 high school students in Hue, Vietnam. Participants were invited to complete a self-reported questionnaire pertaining to individual demographic characteristics, personal perception of self-efficacy, and willingness to perform first aid. To better interpret these findings, both multivariable linear and Poisson regression models were fitted to evaluate the association between individual student characteristics and the willingness to perform first aid.

**Results:**

The prevalence of having willingness to perform first aid (defined as ≥4 points out of 5 to all three questions) was 49.9% (95%CI:28.6–71.2%). The major reported barriers in performing first aid were fear of making mistakes and hurting victims (34.4%, 95%CI:31.9–37.0%), no prior first aid training (29.8%, 95%CI:25.9–33.9%), and forgetting first aid steps (23.0%, 95%CI:15.8–32.2%). By employing the multivariable linear regression model, it was identified that students with high (β = 0.614, 95%CI:0.009–1.219) or very high (β = 1.64, 95%CI:0.857–2.422) levels of self-efficacy appeared to be more willing to perform first aid. Similarly, in the Poisson regression models, compared to neutral students, students who reported high (PR = 1.214, 95%CI:1.048–1.407) or very high (PR = 1.871, 95%CI:1.049–3.337) levels of self-efficacy were more willing to perform first aid.

**Conclusions:**

The level of willingness to perform first aid among high school students in this study population was found to be moderate. Therefore, integrating activities to promote self-efficacy in first aid training could be considered a progressive step towards improving a student’s willingness to provide such life-saving procedures.

## 1. Introduction

Child and adolescent injuries are a significant cause of childhood mortality globally, leading to a large burden upon public health in low- and middle-income countries (LMICs) [1,2]. According to a study investigating the global burden of diseases in 2017, it was found that injuries are a major factor that is attributed to approximately 1900 child death cases per day worldwide [3]. Recent studies demonstrated that various first-aid interventions such as cardiopulmonary resuscitation (CPR), management of a suspected spinal/head injury or bleeding that is performed by untrained personnel (e.g., caregiver, bystander) or a trained provider could be crucial to mitigate mortality and the risk of disability caused by injuries [4]. Additionally, training children in first aid techniques has been considered a novel strategy to increase the rate of CPR within a community, thus resulting in higher survival rates [5–7]. A high level of willingness to perform first aid among adolescent bystanders can improve the timely access to quality care for victims, contributing to the reduction of morbidity and mortality caused by injuries [5,8]. Therefore, the World Health Organization has recommended the “Kids save lives” statement to emphasize the role of children in increasing the rate of CPR amongst lay people [8,9]. While studies of children receiving first aid training have been well documented in developed countries [10,11], the data in developing countries remains limited and presently illustrates a low rate of first aid training for children [12,13]. Regardless, even among children who received first-aid training, their willingness to perform first aid was influenced by various factors. Furthermore, several studies have indicated that the factors related to the lack of students’ willingness to perform first aid include insufficient knowledge [11], fear of hurting the victim [6], and the fact that the victim was a stranger [11].

In a lower-middle-income country such as Vietnam with 26.2 million children (i.e., 28.0% of the population in 2017) [14], data on the willingness to perform first aid across children remains poorly reported [15,16], despite the importance of willingness to perform first aid [6,11,17–19]. Vietnamese children have been suffering from a large burden attributed to injuries such as fall, motor vehicle accident, and being attacked or abused or fighting with someone [20]. In 2017, injuries led to 7400 excessive deaths in Vietnamese children aged 10–19 years old [21]. A recent Vietnamese national survey in 2013, which included high school students, indicated that 34.3% of boys and 25.1% of girls were injured at least once during the past year [20]. Though schools are encouraged to organize first aid training for teachers, staff, and students, there remains a lack of standardized formal curriculum at both regional and national levels in Vietnam [22].

To our knowledge, there are no published datasets that assess the willingness of students to perform first aid and associated factors in Vietnam. Therefore, it is essential to conduct studies reflecting the present efficacy of first aid training and recommend means to bolster training quality. Thus, this study aims to estimate the prevalence of willingness to perform first aid amongst high-school students in Hue and thus identify factors that may be associated with first aid interventions.

## Methods

### Study design and study population

A cross-sectional study on high school students in Hue was conducted from April to July 2020. Hue is a major education and healthcare center within Vietnam, with an estimated population of 652,572 in 2021 [23]. All high schools in Hue, in addition to other provinces in Vietnam, are required to follow a national framework provided by the Vietnamese Ministry of Education’s general education program [24]. General education comprises three levels: five years of primary education (elementary school), four years of lower secondary education (middle school), and three years of upper secondary education (high school) [25].

However, as most students in grade 12 tend to have busy schedules in order to prepare for the National High School Exam, we decided to select grades 10 and 11 students, who have more flexible schedules from high schools across Hue as the source population. There are presently 11 high schools in Hue [26], with 8328 high school students within grades 10 and 11 at the time of the study.

### Sample and selection of participants

Data were collected during the baseline assessment phase of the first aid training project of Hue University of Medicine and Pharmacy. The required sample size for this study was calculated by utilizing a formula for cross-sectional studies [27] with 95% confidence level and 5% precision and accounted for the proportion of participants who have demonstrated sufficient first aid knowledge p = 0.491, as assessed in a previous study [28]. Due to the use of a multistage stratified random sampling design, the design effect was multiplied by a factor of 2. To account for participants refusing to complete or returning incomplete questionnaires, an additional 10% of the sample population was added. Overall, 844 students were included in the study population.

**Fig 1:**
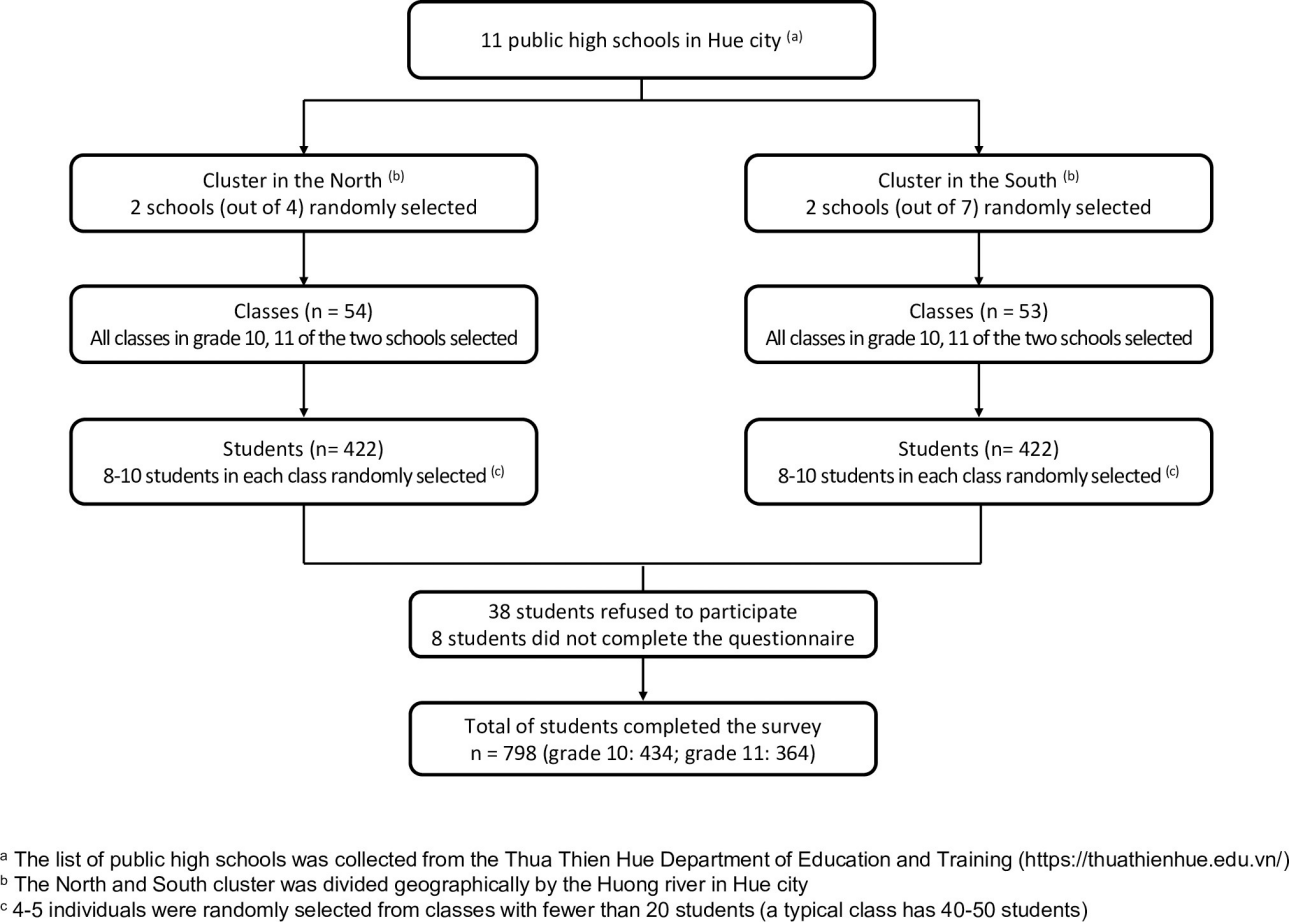
Flow chart of participants selected in this study.

A multistage stratified random sampling technique was utilized to produce a representative sample of high school students from Hue. 11 public high schools within Hue were stratified into two groups, north or south schools that are separated by the Huong River. At the first stage, we randomly selected four high schools (two schools in the north and two schools in the south of the city) (Fig 1). At the second stage, among 4 selected representative high schools, all classes in grades 10 and 11 were selected. Approximately 8–10 students were randomly selected in each class. We collaborated with the schools to meet and obtain agreement from the selected students and their parents. Students were asked to complete a self-reported electronic-based questionnaire on smartphones, tablets, or laptops under the research staffs’ in-person instructions. In the case of students who did not own an electronic device to get access to the questionnaire, we would provide our tablets.

The inclusion criteria for participants included being in grade 10 or grade 11 at selected high schools during recruitment, receiving permission to participate in the study from parents or legal guardians, and having both physical and mental capacity to answer the questionnaire of the study.

### Instrument development

After conducting the literature review, we developed a questionnaire to investigate the prior acquisition of first aid training, current levels of self-efficacy, and willingness to perform first aid (S3 Table). All selected questions were translated into Vietnamese and back-translated to English using the approach of Back-Translation for Cross-Cultural Research [29]. The translation process includes the following steps: forward translation, back-translation, back translation review and discussion by the expert panel, and finalization. A language expert, a senior lecturer in the nursing department, translated all English questions into Vietnamese. Another independent senior emergency physician back-translated those questions into the English version. The consensus panel was then carried out to ensure the two English versions were comparable. The questions had modest adjustments to fit Vietnamese culture and language throughout the translation procedure. According to Bandura (1997), “Perceived self-efficacy is concerned with people’s beliefs in their capabilities to produce given attainments” [30]. From this definition, we defined self-efficacy as the participant’s belief in their capabilities to perform first aid and save human lives. According to Bandura’s Self-Efficacy Theory, we constructed the self-efficacy scale for first aid [30]. Based on a previous study by Wei et al. in 2013 [31] which discussed the common types of injury among Vietnamese high school students [32], we decided to use a self-efficacy score calculated on the basis of six questions. The participants were then asked to rank personal levels of self-efficacy to perform each of the six essential first aid skills (including calling emergency, cardiopulmonary resuscitation, chest compression, mouth-to-mouth ventilation, immobilization of fracture, and stopping bleeding). Point for each skill could range from 1 point as “Not confidence at all” to 5 points as “Very confident.” The maximum possible score which denotes the highest level of self-efficacy for all six first aid skills was 30, a minimum score of 5 illustrates the lowest level of self-efficacy. Apart from the continuous scale, we classified the student’s self-efficacy into 5 categories as very low (<10 points), low (10–14 points), neutral (15–19 points), high (20–24 points), and very high (≥25 points).

Willingness to perform first aid by students was calculated based on three questions in a similar ranking system which was previously described [33]. Questions inquiring about self-reported levels of participant willingness to perform first aid was divided into three different circumstances: (1) a victim is a stranger, (2) the student is the only person who can help in the accident, and (3) other people are also present in the accident were used. For each of the three questions, participants were asked to rank their level of willingness to perform first aid as “fully disagree” (1 point) to “fully agree” (5 points). The maximum score of willingness is 15, and the minimum score is 3. We defined persons as being willing to perform first aid if their willingness mean score was higher than the mean score of the study population; otherwise, they would be classified as not willing to perform first aid. Also, we presented results using another definition of being willing to perform first aid which required a person to respond “agree” or “fully agree” (≥ 4 points, i.e., only positive responses) in all three willingness questions; otherwise, they would be categorized as not willing to perform first aid.

To evaluate the content and face validity of the presented questions, a panel of experts on nursing and specialists in emergency medicine at the Emergency Department at Hue University of Medicine and Pharmacy was established. The expert panel commented and approved the first draft of the survey which included questions on general characteristics (sex, age, school, class), history of injuries (number of injuries, types of most serious injury, causes of the injury), any experience with first aid training, self-efficacy, and willingness to provide first aid. Each individual item in the draft questionnaire was evaluated for readability, appropriateness, concreteness, and significance. After that, two researchers discussed the questionnaire with six high school students from Hue to collect their feedback on the readability and adequacy of the questionnaire. A quantitative pilot survey was also implemented on 50 students from the source population to create an exhaustive list of choices for the final multiple-choice questions. The time estimated to complete the questionnaire is about 20 minutes. Ultimately, the expert panel finalized the revised questionnaire which was then employed for this study.

### Data analyses

In terms of construct validity, Exploratory Factor Analysis (EFA) was carried out to identify the possible latent variables that uncover the structure of items in questionnaire. First, we established the model using principal component analysis, followed by a scree plot [34] and parallel analysis [35] to determine the number of factors. The following criteria were used to identify the number of factors: a) eigenvalues larger or equal to the eigenvalue at the scree plot’s "elbow", and (b) explain more than 80% of the total variation.

After the number of factors was defined, we carried out the iterative principal factor analysis with an oblique Promax rotation method to extract factors. The items with the highest loading factor <0.4, or uniqueness >0.5 would be removed. The procedure was repeated until no more objects in the model were removed. The results of Bartlett’s test of sphericity [36] and the Kaiser-Meyer-Olkin (KMO) measure of sample adequacy [37] were used to evaluate the applicability of EFA. The Cronbach’s Alpha was also used to measure internal consistency.

All statistical analyses accounted for the multiple-stage sampling strategy with the “svy” options in Stata version 15.1 [38]. Sampling weights were employed to obtain representative estimates of the whole population of high school students in Hue, Vietnam and to adjust for non-response bias and over or under-sampling.

Weighted proportions and confidence intervals were calculated for categorical variables, the weighted mean and confidence intervals were computed for continuous variables. Univariate comparisons on participant demographics, prior first aid training, self-efficacy, and willingness were assessed using Pearson Chi-Squared, Fisher’s exact tests, or independent t-test.

To identify associated factors, we conducted a literature review and created a simple causal diagram (DAG—directed acyclic graph) to illustrate the relationship between variables [39–41]. VanderWeele et al. and Hernán et al. suggested that this approach would provide more valid estimates as compared to traditional biostatistical approaches, such as backward and forward selection [39–41].

For the continuous score of willingness, we fitted a multivariable linear regression model to explore the association between the willingness to perform first aid and the student’s personal characteristics such as their level of self-efficacy, prior first aid training (Yes/No), class, sex, and injury experience in the past 12 months (Yes/No) which required participants to be absent from school for at least one day. We also examined the associated factors with willingness to perform first aid categorized by average mean score or positive responses as described in section 2.3 using Poisson regression models with binary outcomes. As the prevalence of students who lack the willingness to perform first aid among the study population was greater than 10%, the association between these binary outcomes and independent variables would be overestimated using logistic regression. Therefore, using log-binomial regression models to directly approximate Prevalence Ratios (PRs) would be an alternative solution; however, this model often fails to converge [42]. To tackle these statistical issues, previous studies calculated PRs by employing a modified Poisson regression model with a robust error variance for binary outcome data [43,44]. With such a model, Chen et al. demonstrated that the results were comparable to log-binomial regression models [45].

After data analysis in Stata 15.1, R statistical software version 3.4.0 and the packages ggplot2 and ggpubr [46,47] were used to visualize the study’s findings.

### Ethical considerations

Our research proposal was approved by the Institutional Review Board (IRB) of Hue University of Medicine and Pharmacy with the registration number: H2020/057. We also obtained permission from the executive boards of all four selected high schools. All participants and their parents were informed of the purpose of the study and explained the minimal risks involved with the participation and the confidentiality of their data. All participation was voluntary, and the respondents could quit the study whenever they wanted. All written consent forms were collected before students participated in the study. According to the Vietnamese law of children, children are defined as under 16 years old [48]. As all eligible participants in this study were 16 years old and above, they are not considered children or minors. Therefore, consent from parents or guardians was not required by the local IRB at Hue University of Medicine and Pharmacy. However, the research team did get verbal consent from the guardians of eligible students. After completing the questionnaire, every student received a reusable water bottle as a small gift as compensation for their time.

## Results

In total, 844 students were randomly invited to the study; among this group, only 798 students (94.5%) agreed to participate and completed the questionnaire (Fig 1).

### Study population characteristics

Table 1 shows the weighted characteristics of study participants by sex. Approximately half of the study participants were in grade 10. With regards to first aid training, only 9.1% (95%CI: 1.5–38.7%) of female students had ever attended a first aid training course, while this proportion in male counterparts was 13.6% (95%CI: 5.6–29.4%); however, this difference was not statistically significant (p = 0.159). Among those trained in first aid, only about ⅓ of participants were trained within the past year across both sexes. Participants mainly received information detailing first aid procedures from the internet (84.8%, 95%CI: 60.5–95.3%) and their teachers (63.5%, 95%CI: 35.4–84.6%). Over 15% of students (95%CI: 7.4–29.4%) experienced an injury at least once during the past 12 months.

**Table 1:** The weighted characteristics of students by sex.

Characteristics	Prevalence in females (95%CI) ^a^	Prevalence in male (95%CI) ^b^	Total prevalence (95%CI) ^c^
**Grade**			
10	52.4 (39.7–64.9)	43.9 (37.8–50.3)	49.1 (42.1–56.2)
11	47.6 (35.1–60.3)	56.1 (49.7–62.2)	50.9 (43.8–57.9)
**Prior first aid training**			
No	90.9 (61.3–98.5)	86.4 (70.6–94.4)	89.2 (66.0–97.2)
Yes	9.1 (1.5–38.7)	13.6 (5.6–29.4)	10.8 (2.8–34.0)
≤ 1 year training	38.4 (8.3–81.1)	33.4 (5.4–81.6)	36 (7.6–79.3)
>1 year training	38.7 (8.0–82.1)	54 (8.5–93.6)	46.2 (9.8–87.1)
Don’t know	22.9 (9.6–45.5)	12.6 (2.6–43.6)	17.8 (6.4–40.7)
**Sources of first aid information if received (Multiple answers)**			
Internet	85.1 (67.9–93.9)	84.3 (44.4–97.3)	84.8 (60.5–95.3)
Facebook	55.9 (34.6–75.3)	49.9 (30.5–69.3)	53.6 (34.8–71.4)
Relatives	52.1 (30.2–73.2)	41.6 (19.8–67.3)	48 (26.7–70.0)
Friends	36.2 (31.6–41.1)	34.1 (26.8–42.4)	35.4 (29.8–41.5)
Television	56.8 (35.8–75.5)	51 (17.7–83.5)	54.5 (27.8–78.9)
Teachers	69.3 (36.4–89.9)	54.4 (31.8–75.3)	63.5 (35.4–84.6)
Others (movies, books…)	2.7 (0.5–12.6)	4.8 (2.0–11.3)	3.6 (1.5–7.9)
**Injury experience in the past 12 months**			
No	86.1 (70.2–94.2)	82.3 (69.6–90.4)	84.6 (70.6–92.6)
Yes	13.9 (5.8–29.8)	17.7 (9.6–30.4)	15.4 (7.4–29.4)

^a^ Weighted N = 5065.

^b^ Weighted N = 3263.

^c^ Weighted N = 8328.

**Fig 2:**
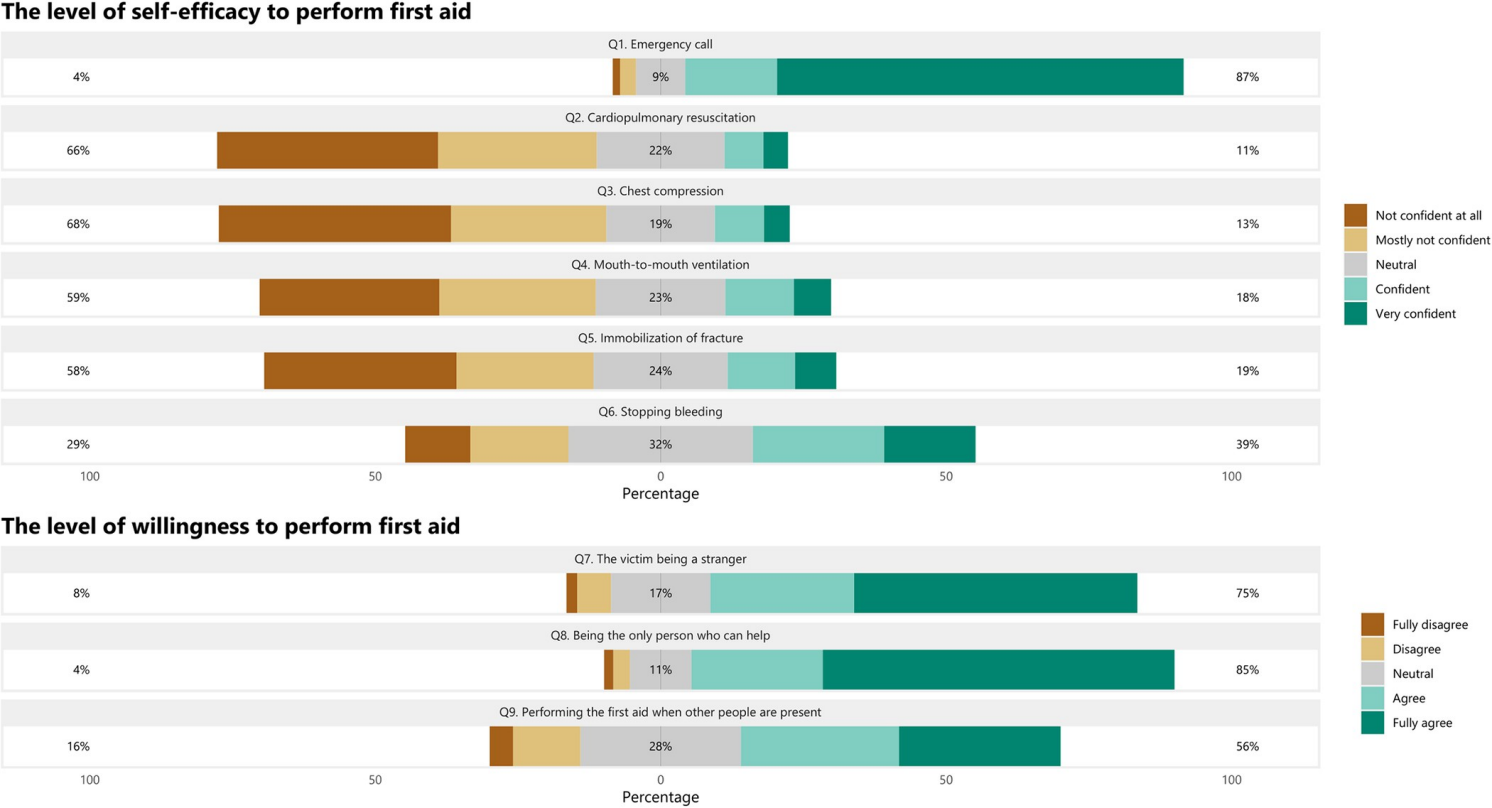
The self-efficacy and willingness of students regarding first aid.

Fig 2 presents the weighted proportions of responses for each question regarding levels of self-efficacy and the willingness to perform first aid. Among the six first aid skills asked, participants reported low confidence for the administration of most first aid skills, except for calling for emergency services. However, with regards to willingness in the three first aid circumstances, participants mostly agreed to provide first aid, especially when they were the only person who could help victims (84.6%, 95%CI: 70.2–92.7%), as well as when the victim was a stranger (74.8%, 95%CI: 53.6–88.4%). Approximately half of the participants were willing to engage in emergency scenarios when other bystanders were also present (56%, 95%CI: 30.7–78.5%).

### Self-efficacy of students regarding first aid

In terms of self-efficacy of students regarding first aid, the preliminary EFA model revealed that three components were sufficient (S1 Fig). The refined model appeared to have three factors (S4 Table).

The pattern of factor loading and correlation matrix among the factors of an oblique rotation are presented in S4 and S5 Tables, respectively. The content of the items indicates that these three factors focus on the quick response (Emergency call), basic life support (Cardiopulmonary resuscitation, chest compression, mouth-to-mouth ventilation), and first aid for injury (Immobilization of fracture, stopping bleeding). The results of Barlett’s test and the KMO statistic show that the EFA model was appropriate. The Kaiser-Mayer-Olkin MSA was high (0.816 > 0.5) [49]. Bartlett’s test suggested that the correlation matrix was not random (p < 0.001) [50]. Therefore, our data were suitable for factor analysis.

The Cronbach’s alpha coefficient of self-efficacy scale was 0.8321. This suggests the scale has good internal consistency [51].

Table 2 presents the weighted prevalence of different levels of self-efficacy of first aid. Most students (about 70%) showed a neutral, low, or very low level of first aid self-efficacy. Male students have a significantly higher self-efficacy mean score (17.1, 95%CI: 15.5–18.8) than female students (p-value = 0.006 from t-test). Consistently, the male group tended to have a greater proportion of high (29.3% (95%CI: 22.0–38.0%)) and a very high level of efficacy (6.4% (95%CI: 0.9–34.0%)) in comparison to female students. About 46% of students with prior first aid training reported a high or very high level of self-efficacy, whereas this proportion in those without previous training was only 26%. There was not a statistically significant difference between the levels of self-efficacy between the students who were trained in the past year and those who received training more than one year ago.

**Table 2:** The weighted prevalence of self-efficacy of students regarding first aid.

Characteristics	Self-efficacy score	Level of self-efficacy ^a^				
		Very low (<10 points)	Low (10–14 points)	Neutral (15–19 points)	High (20–24 points)	Very high (≥25 points)
	Mean (95%CI)	% (95% CI)	% (95% CI)	% (95% CI)	% (95% CI)	% (95% CI)
**Overall**	16.6 (15.0–18.2)	4.2 (1.4–11.5)	34.7 (23.9–47.3)	32.6 (29.8–35.6)	22.4 (16.0–30.3)	6.2 (1.1–27.1)
**Sex**						
Female	16.2 (14.6–17.8)	4.7 (1.7–12.6)	36.8 (22.6–53.9)	34.5 (24.8–45.7)	17.9 (12.2–25.5)	6 (0.9–29.8)
Male	17.1 (15.5–18.8)	3.3 (1.0–10.4)	31.3 (26.5–36.6)	29.7 (16.2–47.9)	29.3 (22.0–38.0)	6.4 (0.9–34.0)
p-value	0.006 ^b^	0.195 ^c^				
**Grade**						
10	16.9 (14.9–18.9)	3.4 (1.8–6.3)	33.1 (20.8–48.4)	35.6 (23.0–50.4)	21.3 (16.5–27.2)	6.5 (1.0–32.5)
11	16.2 (14.8–17.7)	4.9 (0.6–28.6)	36.2 (26.6–47.0)	29.8 (20.9–40.6)	23.4 (14.9–34.7)	5.8 (1.2–23.3)
p-value	0.133 ^b^	0.470 ^c^				
**Prior first aid training**						
No	16.4 (14.6–18.3)	4.4 (1.7–10.7)	35.7 (28.0–44.2)	33.6 (29.3–38.2)	20.4 (19.1–21.8)	6 (0.9–31.1)
Yes	17.7 (16.9–18.4)	2.5 (0.1–50.8)	26.6 (3.5–78.5)	24.8 (10.8–47.4)	38.7 (14.1–70.9)	7.4 (1.4–31.3)
p-value	0.117 ^b^	0.237 ^c^				
≤1 year	18 (13.9–22.1)	7 (0.0–92.4)	25.3 (4.7–70.1)	18.6 (8.6–35.5)	36.7 (30.3–43.5)	12.4 (4.6–29.5)
>1 year	18.2 (14.4–21.9)	N/A	22.2 (0.9–90.4)	26.7 (13.5–46.1)	46.5 (8.2–89.4)	4.6 (0.4–38.1)
Do not know	15.7 (15.3–16.1)	N/A	40.5 (10.8–79.3)	32.5 (6.5–76.8)	22.6 (2.2–79.3)	4.4 (0.0–81.7)
p-value	0.945 ^d^	0.415 ^e^				
**Sources of first aid information (Multiple answers)**						
Internet	16.5 (15.3–17.8)	3.3 (1.6–7.0)	35.5 (24.2–48.7)	33.6 (32.9–34.3)	22.2 (14.1–33.0)	5.4 (1.7–16.1)
Facebook	16.3 (15.3–17.3)	3.6 (1.1–11.1)	36.3 (24.1–50.7)	34.8 (28.2–42.2)	20.1 (12.8–30.0)	5.1 (1.6–15.4)
Relatives	16.6 (16.0–17.2)	3.8 (1.1–12.4)	34.5 (19.5–53.4)	34.1 (26.2–43.1)	23.3 (17.0–30.9)	4.4 (1.6–11.6)
Friends	16.9 (15.8–17.9)	4.1 (0.4–29.3)	31.3 (17.9–48.7)	31.8 (19.0–48.0)	27 (9.2–57.5)	5.8 (0.6–37.2)
Television	16.5 (15.5–17.4)	3 (0.9–9.7)	35.7 (23.8–49.7)	34.5 (29.4–39.9)	22.5 (13.9–34.1)	4.3 (0.6–24.0)
Teachers	16.6 (15.4–17.8)	4.2 (0.8–19.3)	33.9 (18.0–54.5)	34.6 (26.3–43.9)	21.5 (16.3–27.9)	5.8 (1.4–21.6)
Others (movies, books…)	19.7 (16.6–22.8)	N/A	17.7 (4.0–52.5)	19.6 (2.8–67.2)	56 (15.2–90.0)	6.8 (0.3–61.0)

Abbreviations: CI, confidence interval; N/A, not applicable as there was no participant in this category.

^a^ The level of self-efficacy was classified based on the sum of scores from six Likert self-efficacy questions.

^b^ p-value from an unpaired t-test.

^c^ p-value from Chi-squared test.

^d^ p-value from an unpaired t-test, the group who answered “do not know” was not included in statistical tests.

^e^ p-value from Chi-squared test, the group who answered “do not know” was not included in statistical tests.

### Willingness of students to perform first aid

Similar to the Self-efficacy scale, we developed the EFA model for items measuring willingness. However, because the willingness scale includes three items, we can define only 1 factor. In addition, the rotation approach is not applied to a single factor. Factor loading of items measuring willingness is shown in the S6 Table.

The results of Bartlett’s test (p < 0.001) and Kaiser-Mayer-Olkin MSA (0.687) indicate that the factor analysis can be useful.

The Cronbach’s alpha coefficient for willingness scale was 0.7649. This indicates that the internal consistency of the self-efficacy items is acceptable [51].

Table 3 indicates that half of all surveyed high school students of both sexes reported that they would be willing to perform first aid skills in all three aforementioned circumstances. The estimated prevalence utilizes two definitions that are similar in each characteristic. Students in grade 10 had a slightly higher mean willingness score as well as a higher prevalence of willingness, but this was found to be not statistically significant (p-value: 0.286, t-test). Interestingly, students without prior first aid training had a slightly higher mean score (12.2 (95%CI: 10.9–13.5)) and higher mean-based prevalence of willingness (54%, 95%CI: 33.9–73.0%), as well as positive response-based prevalence (50.9%, 95%CI: 33.7–68.0) in comparison to students who reported having received first aid training before. However, these differences were not statistically significant. Furthermore, students with a higher level of self-efficacy to perform first aid appeared more willing to administer first aid to the victim. There were no noticeable differences in the mean score or prevalence of willingness across strata of sources of first aid information received.

**Table 3:** The weighted prevalence of willingness of students to perform first aid.

Characteristics	Willingness score	Prevalence of having the willingness to perform first aid	
		Defined based on the mean of willingness score ^a^	Defined based on having positive responses of willingness ^b^
	Mean (95%CI)	% (95%CI)	% (95%CI)
**Overall**	12.2 (10.8–13.6)	52.1 (28.2–75.1)	49.9 (28.6–71.2)
**Sex**			
Female	12.2 (10.8–13.6)	51.7 (28.8–73.9)	50.7 (28.8–72.3)
Male	12.2 (10.5–13.9)	52.7 (25.1–78.7)	48.6 (27.1–70.7)
p-value	0.946 ^c^	0.815 ^d^	0.516 ^d^
**Grade**			
10	12.6 (12.0–13.2)	59 (46.2–70.6)	56.4 (47.4–64.9)
11	11.8 (9.4–14.3)	45.4 (14.5–80.4)	43.6 (14.4–77.9)
p-value	0.286 ^c^	0.175 ^d^	0.203 ^d^
**Prior first aid training**			
No	12.2 (10.9–13.5)	54 (33.9–73.0)	50.9 (33.7–68.0)
Yes	11.9 (10.0–13.8)	36 (8.2–78.1)	40.9 (6.5–87.2)
p-value	0.201 ^c^	0.093 ^d^	0.414 ^d^
≤ 1 year	11.7 (9.9–13.4)	30.6 (27.9–33.5)	45 (31.5–59.3)
>1 year	12.3 (10.1–14.6)	43.8 (4.3–93.2)	41.4 (2.9–94.3)
Do not know	11.1 (6.0–16.2)	26.9 (0.3–97.9)	31.5 (0.6–97.3)
p-value	0.276 ^e^	0.482 ^f^	0.842 ^f^
**Level of self-efficacy**			
Very low (<10 points)	10.4 (8.5–12.4)	27.5 (3.2–81.2)	17.2 (7.2–35.5)
Low (10–14 points)	11.7 (9.1–14.3)	48.1 (23.5–73.8)	45.2 (22.6–70.0)
Neutral (15–19 points)	12.2 (11.7–12.7)	49.4 (28.1–71.0)	47.9 (32.1–64.1)
High (20–24 points)	12.7 (11.5–14.0)	58.1 (34.4–78.6)	55.6 (26.7–81.1)
Very high (≥25 points)	13.8 (13.4–14.2)	83.1 (61.9–93.7)	87.8 (40.4–98.7)
p-value	0.066 ^f^	0.063 ^d^	0.022 ^d^
**The sources of first aid information if received (multiple answers)**			
Internet	12.2 (10.8–13.7)	52.5 (27.1–76.7)	50.4 (29.0–71.7)
Facebook	12.2 (10.9–13.4)	51.1 (25.6–76.0)	49.1 (27.5–71.0)
Relatives	12.4 (10.9–13.8)	55.5 (28.4–79.7)	52.8 (34.1–70.8)
Friends	12.3 (10.4–14.2)	52.6 (14.9–87.5)	50.5 (22.7–78.1)
Television	12.3 (11.2–13.4)	52.5 (31.9–72.3)	50.3 (34.7–65.8)
Teachers	12.4 (11.9–12.9)	52.5 (34.4–70.0)	51.3 (34.0–68.2)
Others (movies, books…)	12.8 (11.9–13.7)	59.7 (30.7–83.2)	55 (42.6–66.8)

Abbreviations: CI, confidence interval.

^a^ Having willingness to perform first aid was defined as having the willingness score larger than the mean of willingness score of the study population (from 12.2 points and above).

^b^ Having willingness to perform first aid was defined as having positive responses (i.e., responded “agree” or “fully agree”) in all three Likert willingness questions.

^c^ p-value from an unpaired t-test.

^d^ p-value from Chi-squared test.

^e^ p-value from an unpaired t-test, the group who answered “do not know” was not included in statistical tests.

^f^ p-value from Chi-squared test, the group who answered “do not know” was not included in statistical tests.

### Barriers and facilitating factors associated with students’ willingness to perform first aid

**Fig 3:**
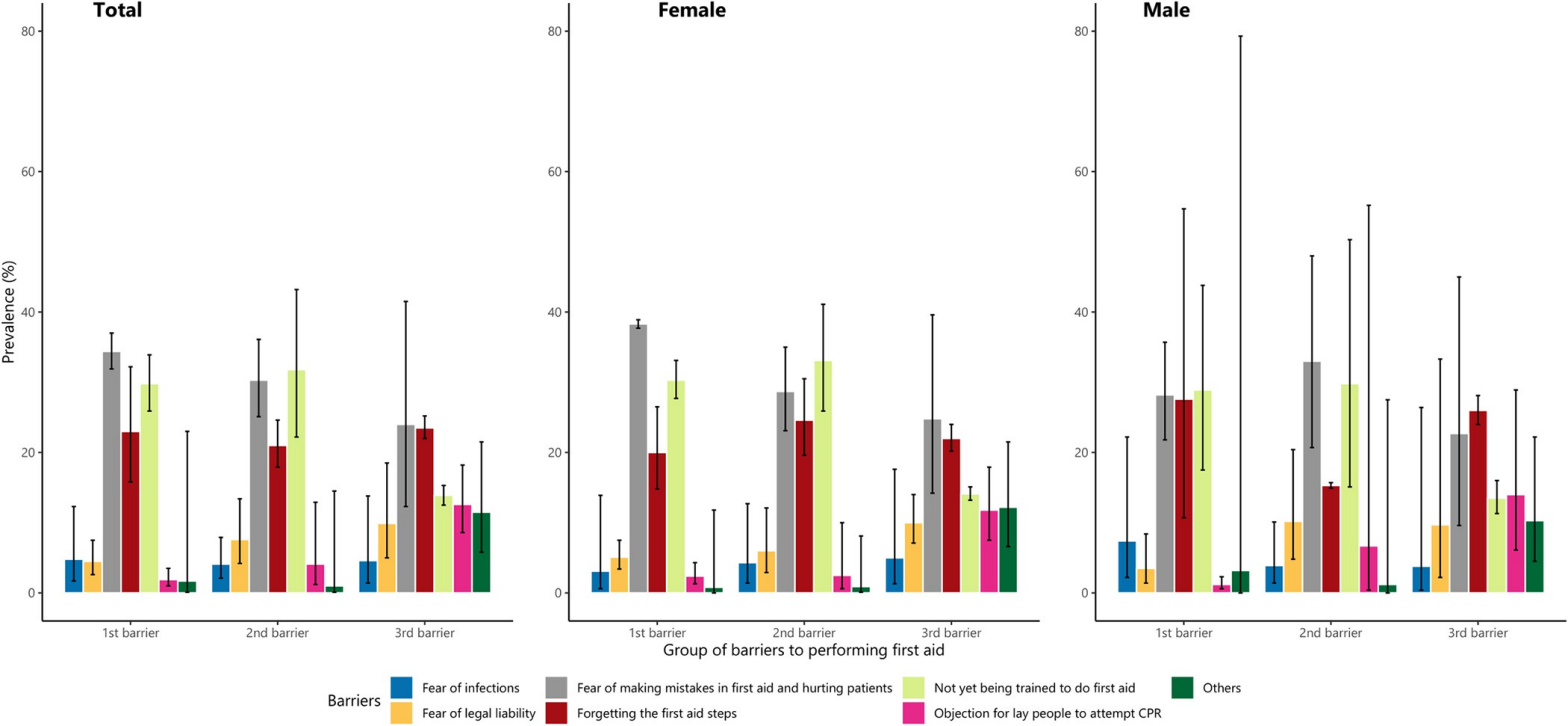
The barriers associated with the students’ willingness to perform first aid.

We asked students to rank the top three barriers which may prevent them from providing first aids. The proportion of students choosing each barrier as the first, second, and third barrier by sex was presented in Fig 3. The three barriers that surfaced in all first, second, and third rank were fear of making mistakes and hurting victims (34.1%, 95%CI: 31.3–37.1), not yet been trained to do first aid (30.3%, 95%CI: 28.3–32.4), and forgetting first aid steps (22.9%, 95%CI: 15.5–32.3) (Fig 3). Fear of making mistakes and hurting victims was more common in female students (38.2%, 95%CI: 37.6–38.8) as compared to male students (27.8%, 95%CI: 21.2–35.6) (S1 Table).

**Fig 4:**
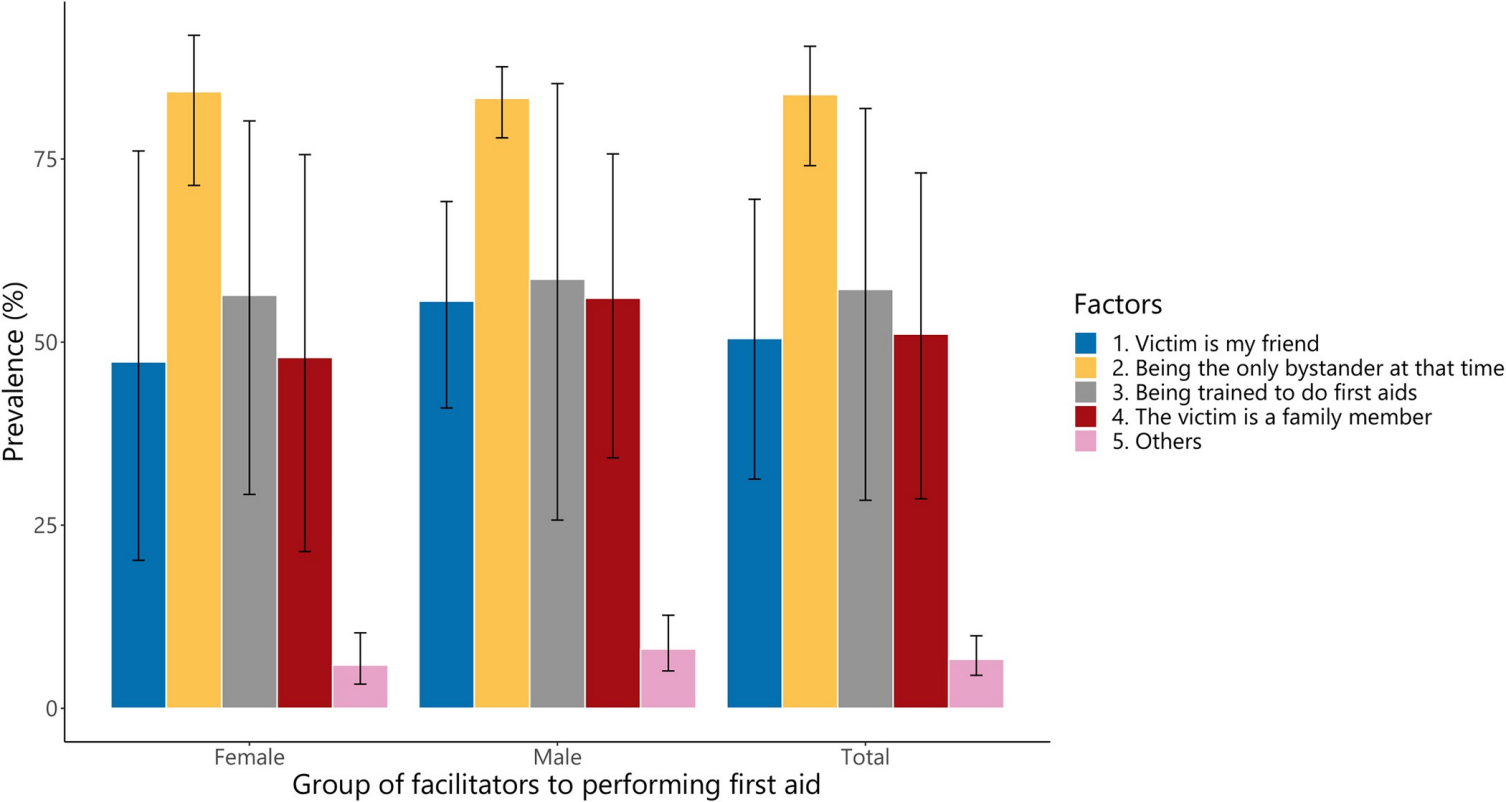
The facilitating factors associated with the student’s willingness to perform first aid.

Fig 4 shows the proportions of students who responded “Yes” to facilitating factors listed in the questionnaire. The most common factor that motivated high school students to perform first aid was being the only bystander in accident circumstances (83.9%, 95%CI: 74.1–90.5%), followed by “being trained to do first aids” (57.6%, 95%CI: 27.7–82.8%). The proportions of facilitating factors were not different between males and females (p = 0.683, Chi-square test) (S2 Table).

### Factors associated with students’ willingness to perform first aid

Based on the multivariable models from Table 4, we found that the level of self-efficacy and prior first aid training was significantly associated with students’ willingness to perform first aid.

**Table 4:** The multivariable model of students’ willingness to perform first aid.

Willingness score	Model 1^a^ (Continuous scale)		Model 2^b^ (Dichotomized by mean)		Model 3^c^ (Dichotomized by positive responses)	
	β	95% CI	PR	95% CI	PR	95% CI
**Levels of self-efficacy**						
Very low (<10 points)	**-1.706**	**((-3.023)–(-0.39))**	0.57	(0.122–2.67)	0.367	(0.105–1.282)
Low (10–14 points)	-0.481	((-2.481) - 1.518)	0.991	(0.713–1.377)	0.959	(0.777–1.183)
Neutral (15–19 points)	Ref	-	Ref	-	Ref	-
High (20–24 points)	**0.614**	**(0.009–1.219)**	1.236	(0.946–1.617)	**1.214**	**(1.048–1.407)**
Very high (≥25 points)	**1.64**	**(0.857–2.422)**	**1.718**	**(1.161–2.541)**	**1.871**	**(1.049–3.337)**
**Prior first aid training**						
No	Ref	-	Ref	-	Ref	-
Yes	**-0.518**	**((-0.899)–(-0.136))**	0.629	(0.311–1.274)	0.762	(0.314–1.848)
**Sex**						
Female	Ref	-	Ref	-	Ref	-
Male	-0.008	((-1.335) - 1.32)	1.029	(0.707–1.498)	0.959	(0.72–1.279)
**Injury experience in the past 12 month**						
No	Ref	-	Ref	-	Ref	-
Yes	-0.435	((-2.514) - 1.644)	1.004	(0.575–1.755)	0.96	(0.483–1.91)
**Grade**						
10	Ref	-	Ref	-	Ref	-
11	-0.725	((-2.786) - 1.336)	0.775	(0.409–1.467)	0.783	(0.4–1.533)

Abbreviations: PR, prevalence ratio calculated from Poisson regression model; CI, Confidence interval.

Bold numbers indicated statistically significant results with p figure <0.05.

^a^ Model 1: Multivariable linear regression model with the continuous willingness score (ranging from 3 to 15 points) as the outcome.

^b^ Model 2: Multivariable Poisson regression model with the binary willingness variable as the outcome. Having willingness to perform first aid was defined as having the willingness score greater than the mean of willingness score of the study population (from 12.2 points and above).

^c^ Model 3: Multivariable Poisson regression model with the binary willingness variable as the outcome. Having willingness to perform first aid was defined as having positive responses (i.e., responded “agree” or “fully agree”) in all three Likert willingness questions.

In the multivariable linear regression model, compared to neutral students, students with a very low level of self-efficacy were less willing to perform first aid (β = -1.706, 95%CI: (-3.023)—(-0.39)); whereas, those with a high or very high levels of self-efficacy were significantly more willing to do such techniques (β = 0.614, 95%CI: 0.009–1.219; β = 1.64, 95%CI: 0.857–2.422, respectively). Additionally, any prior first aid training experience had a significant negative association with students’ willingness (β = -0.518, 95%CI: (-0.899)–(-0.136)).

In the multivariable Poisson regression models, only high and very high levels of self-efficacy were associated with having willingness to perform first aid, as compared to students with neutral self-efficacy. The factor of previously receiving first aid training was not statistically significantly associated with willingness in these two models.

## Discussion

In the present study, the willingness to perform first aid among high school students within a Vietnamese city was approximately 50%; this is lower than studies conducted across high school students in China (73%) [13], Hong Kong (83.3%) [52], New Zealand (63%) [53], and Japan (50–68.2%) [11]. However, the finding of this study is slightly higher than the results reported in Malaysia with 45.1% [54]. Overall, these differences could be due to variation in specific questionnaires and definition of willingness to perform first aid between studies. For example, one Japanese study utilized dichotomous questions on five hypothetical scenarios of cardiopulmonary arrest to estimate the prevalence of willingness to perform first aid among students [11]. Furthermore, other studies in New Zealand and China employed Likert questions which primarily focus on two scenarios, such as if the victim was a stranger or family member [13,53].

This study also investigated the leading factors which act as barriers or facilitators in influencing the willingness of students to perform first aid. The presented study further illustrates that the fear of making mistakes and hurting victims (38.2%, 95%CI: 37.6–38.8%) remains the most prevalent reason which prevents students from offering first aid. This finding is in line with studies conducted in other countries. For example, in Japan, the reason for unwillingness to perform CPR was the fear of inadequate performance in first aid [11]. Similarly, in Hong Kong, nearly 30% of students reported that being afraid of making mistakes and hurting victims could be a major barrier to performing first aid [52]. A similar finding was found in Malaysian students [54]. Another significant barrier for performing first aid was not receiving first aid training yet (30.4%, 95%CI: 27.6–33.2%). This result was similar to a previous study on Hong Kong high school students who reported that not being trained in first aid was the second most common reason for the reluctance to perform first aid [52].

In our study, only 9.1% of students had experienced first aid training. This prevalence was slightly lower than Hong Kong (12.3%) [52], Malaysia (17%) [54], and far below some highly developed countries such as Japan (59%) [11], New Zealand (70%) [53] where first aid training courses have been formally provided in the education system. In Norway, first aid training has become a compulsory part of the national high school curriculum, with 90% of the Norwegian population receiving at least one first aid training course within ten years [10]. On the other hand, the most prominent motivating factor to perform the first aid was the realization that one is the only one available to provide the help. This study finding was also found in another study conducted in Malaysia [54].

Apart from knowledge of first aid, self-efficacy plays an important role in initiating, maintaining, and changing first-aid behavior [33]. For example, individuals who lack self-efficacy were less likely to adopt first aid knowledge in a real situation. Recent evidence has also indicated that self-efficacy is a significant factor which influences willingness to perform first aid [19,33]. In this study, a low prevalence of students (11.2%) with a high or very high level of self-efficacy in performing first aid is reported (Table 2). This result is slightly higher than results observed within the Japanese public (9%) [19]. However, it is lower than another study in Norway, where this percentage accounts for 57% [33].

After adjusting for school grade, sex, injury experience, first aid training experience, and self-efficacy, it was found that student willingness to perform first aid is associated with levels of self-efficacy in all three regression models. Student groups with a very high level of self-efficacy were more willing to perform first aid, whereas those with low levels tend to be more reluctant to perform the first aid. Self-efficacy is typically utilized to indicate the ability to perform specific actions. In a previous Norwegian study, self-efficacy was the strongest predictor of intended behavior to demonstrate first aid skills [33]. Several studies on the public population of Taiwan and Japan also found a similar finding [19,55]. Although having not received first aid training has been reported as a common barrier to performing first aid, the role of this factor is not clear when analyzing multivariable models. In the linear regression model, we found that students who received first aid training were negatively associated with willingness to perform the first aid; however, this association was not significant in the Poisson regression models. This finding should be interpreted with caution as few studies have evaluated the relationship between self-efficacy and the willingness of students to perform first aid. The results derived from the linear regression model utilized in this study were inconsistent with previous studies where first aid training was found as a significantly positive factor related to the willingness of the public to perform some first aid skills such as CPR [19,56]. However, another study identified that students who received the first aid training showed a lower score of attitudes toward first aid behavior than untrained ones [55], and one study reported that half of the students trained once in first aid were more likely to be afraid of attempting CPR [57]. Furthermore, having prior first aid training was modestly showed to decrease the willingness score by 0.518. While this is statistically significant in the linear regression model, it was not showed to have any association in the Poisson models. Moreover, although we acquired information of experience in first aid training, we did not collect other important factors that may influence the attempt of first aid including the type of training, quality of training, and the frequency of training. Therefore, further investigation is needed to answer these questions.

There are some strengths in this study. First, we employed the multi-stage stratified random sampling approach to select the study participants. Therefore, the presented results are likely representative of the whole high school student population in Hue. Second, to our knowledge, this is the first and largest study on student’s willingness to perform first aid skills in Southeast Asia. Third, apart from CPR skills, our survey covered other first-aid skills including stopping bleeding, immobilizing fractures, and calling emergency services, which have not been well reported in the literature.

This study also has some limitations which need to be considered. First, the study was a cross-sectional study that was unable to establish a causal relationship. Second, the study population included students who are primarily living in urban areas. Therefore, the interpretation of these results upon students living in rural areas needs to be taken with caution. Third, there were a few classes that were under or oversampled as compared to our initial target. Though we applied post-stratification weights in all surveyed analyses to partly adjust for this issue, there would still be residual bias due to sampling. Finally, as there are no international standard questionnaires which have been developed to evaluate the willingness to perform first aid at the time of this study, utilization of the self-developed instrument in this study could have led to challenges to compare the levels of willingness between countries. Moreover, our questionnaire may not have covered all aspects of this issue and potentially overlooked some key factors which influence the willingness of respondents, thus leading to potential biases in our model.

## Conclusions

The willingness of high school students to perform first aid in Hue, Vietnam, was moderate. The most prominent factor for the willingness of students to perform first aid as an intervention remains as individual self-efficacy. The essential integration of boosting self-efficacy in first aid training can be an important aspect to reform first aid training in Vietnam. Further studies are required to explore approaches to improve both willingness, self-efficacy, and knowledge of first aid approaches in Vietnamese children.

## Supporting information

S1 Fig The parallel analysis of efficacy scale. (TIF)

S1 Table Group of barriers to performing first aid among high school students. (DOCX)

S2 Table Group of facilitators to performing first aid among high school students. (DOCX)

S3 Table Literature review for questionnaire. (DOCX)

S4 Table Factor loading of items in the self-efficacy scale. (DOCX)

S5 Table The correlation matrix of the factors after the oblique rotation. (DOCX)

S6 Table Factor loading of items in the willingness scale. (DOCX)
