# Supplementary figures and images for: The willingness to perform first aid among high school students and associated factors in Hue, Vietnam
Source: PLoS One. 2022 Jul 27;17(7):e0271567. doi: 10.1371/journal.pone.0271567 (PMC9328566; doi:10.1371/journal.pone.0271567)

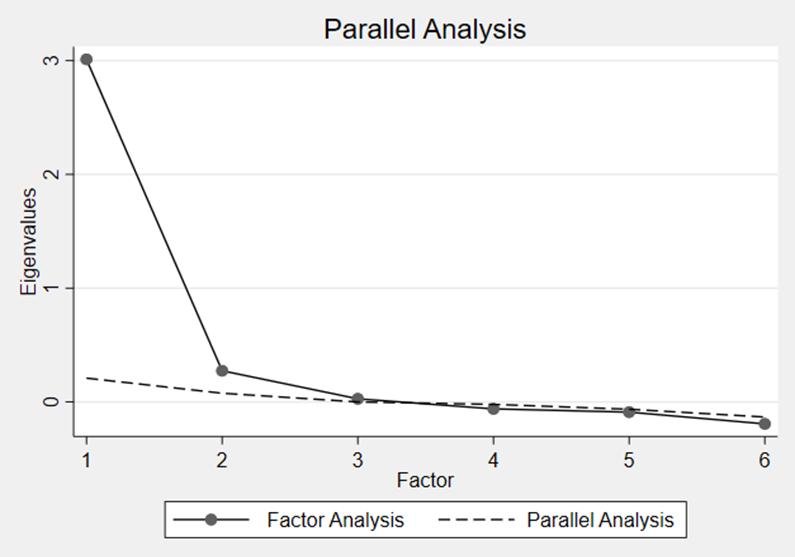

Supplement: S1 Fig — (TIF) [file pone.0271567.s001.tif]
