# Supplementary material for: The willingness to perform first aid among high school students and associated factors in Hue, Vietnam
Source: PLoS One. 2022 Jul 27;17(7):e0271567. doi: 10.1371/journal.pone.0271567 (PMC9328566; doi:10.1371/journal.pone.0271567)
Supplement: S1 Table — (DOCX) [file pone.0271567.s002.docx]

**S1 Table. Group of barriers to performing first aid among high school students**

|  | Female | | Male | | Total | | p value |
| --- | --- | --- | --- | --- | --- | --- | --- |
|  | % | 95%CI | % | 95%CI | % | 95%CI |  |
| **The most important factor** | | | | | | | |
| Forgetting the first aid steps | 20.1 | (14.7-26.8) | 27.1 | (10.8-53.4) | 22.9 | (15.5-32.3) | p = 0.263 |
| Fear of infections | 3.1 | (0.6-13.9) | 7.2 | (2.1-21.6) | 4.7 | (1.8-11.9) |  |
| Fear of legal liability | 5.1 | (3.4-7.5) | 3.4 | (1.5-7.8) | 4.4 | (2.7-7.2) |  |
| Not yet being trained to do first aid | 30.4 | (27.6-33.2) | 30.1 | (21.6-40.2) | 30.3 | (28.3-32.4) |  |
| Fear of making mistakes in first aid and hurting patients | 38.2 | (37.6-38.8) | 27.8 | (21.2-35.6) | 34.1 | (31.3-37.1) |  |
| Objection for lay people to attempt CPR | 2.4 | (1.3-4.3) | 1.2 | (0.6-2.5) | 1.9 | (1.0-3.5) |  |
| Others | 0.8 | (0.0-11.8) | 3.1 | (0.0-79.0) | 1.7 | (0.1-22.9) |  |
| **The second important factor** | | | | | | | |
| Forgetting the first aid steps | 24.6 | (19.5-30.6) | 15.2 | (14.8-15.6) | 20.9 | (18.0-24.1) | p = 0.264 |
| Fear of infections | 4.3 | (1.4-12.7) | 3.9 | (1.3-10.7) | 4.1 | (2.1-7.9) |  |
| Fear of legal liability | 6 | (2.8-12.2) | 9.9 | (4.3-21.2) | 7.5 | (4.0-13.7) |  |
| Not yet being trained to do first aid | 33 | (25.8-41.2) | 29.2 | (16.3-46.8) | 31.6 | (22.6-42.1) |  |
| Fear of making mistakes in first aid and hurting patients | 28.7 | (23.1-34.9) | 34 | (21.7-48.9) | 30.8 | (26.7-35.2) |  |
| Objection for lay people to attempt CPR | 2.5 | (0.6-9.9) | 6.5 | (0.4-54.8) | 4.1 | (1.2-12.9) |  |
| Others | 0.9 | (0.1-8.1) | 1.2 | (0.0-28.3) | 1 | (0.1-14.7) |  |
| **The third important factor** | | | | | | | |
| Forgetting the first aid steps | 22 | (20.3-23.9) | 25.4 | (21.4-30.0) | 23.4 | (21.1-25.8) | p = 0.648 |
| Fear of infections | 5 | (1.3-17.6) | 5.5 | (0.4-47.9) | 5.2 | (1.1-21.1) |  |
| Fear of legal liability | 10 | (7.1-13.9) | 9.5 | (2.0-34.7) | 9.8 | (4.9-18.8) |  |
| Not yet being trained to do first aid | 14.1 | (13.0-15.3) | 13.2 | (10.6-16.4) | 13.8 | (12.1-15.7) |  |
| Fear of making mistakes in first aid and hurting patients | 24.8 | (14.2-39.6) | 22.4 | (10.2-42.5) | 23.9 | (12.6-40.6) |  |
| Objection for lay people to attempt CPR | 11.8 | (7.5-18.0) | 13.8 | (6.1-28.2) | 12.6 | (8.5-18.2) |  |
| Others | 12.2 | (6.6-21.5) | 10.1 | (4.0-23.0) | 11.4 | (5.6-21.9) |  |
