# Supplementary material for: The willingness to perform first aid among high school students and associated factors in Hue, Vietnam
Source: PLoS One. 2022 Jul 27;17(7):e0271567. doi: 10.1371/journal.pone.0271567 (PMC9328566; doi:10.1371/journal.pone.0271567)
Supplement: S2 Table — (DOCX) [file pone.0271567.s003.docx]

**S2 Table. Group of facilitators to performing first aid among high school students**

|  | Female | | Male | | Total | | p value |
| --- | --- | --- | --- | --- | --- | --- | --- |
|  | % | 95%CI | % | 95%CI | % | 95%CI |  |
| Victim is my friend | | | | | | | |
| No | 52.8 | (23.9-79.9) | 43.7 | (29.1-59.6) | 49.2 | (29.2-69.5) | p = 0.362 |
| Yes | 47.2 | (20.1-76.1) | 56.3 | (40.4-70.9) | 50.8 | (30.5-70.8) |  |
| Being the only bystander at that time | | | | | | | |
| No | 15.8 | (8.1-28.7) | 16.4 | (11.8-22.5) | 16.1 | (9.5-25.9) | p = 0.683 |
| Yes | 84.2 | (71.3-91.9) | 83.6 | (77.5-88.2) | 83.9 | (74.1-90.5) |  |
| Being trained the first aid skills | | | | | | | |
| No | 43.6 | (19.7-70.9) | 40.6 | (13.1-75.6) | 42.4 | (17.2-72.3) | p = 0.512 |
| Yes | 56.4 | (29.1-80.3) | 59.4 | (24.4-86.9) | 57.6 | (27.7-82.8) |  |
| The victim is a family member | | | | | | | |
| No | 52.1 | (24.3-78.7) | 43.3 | (22.5-66.7) | 48.6 | (25.8-72.1) | p = 0.296 |
| Yes | 47.9 | (21.3-75.7) | 56.7 | (33.3-77.5) | 51.4 | (27.9-74.2) |  |
| Others | | | | | | | |
| No | 94.1 | (89.7-96.7) | 92.1 | (87.0-95.3) | 93.3 | (90.3-95.5) | p = 0.232 |
| Yes | 5.9 | (3.3-10.3) | 7.9 | (4.7-13.0) | 6.7 | (4.5-9.7) |  |
