# Supplementary material for: The willingness to perform first aid among high school students and associated factors in Hue, Vietnam
Source: PLoS One. 2022 Jul 27;17(7):e0271567. doi: 10.1371/journal.pone.0271567 (PMC9328566; doi:10.1371/journal.pone.0271567)
Supplement: S3 Table — (DOCX) [file pone.0271567.s004.docx]

| **S3 Table. Literature review for questionnaire** | | |
| --- | --- | --- |
| **No** | **Questions** | **References** |
| A | General information | |
| A1 | Gender (Male/ Female) |  |
| A2 | Age |  |
| A3 | School class |  |
| B | Experience of first aid training | |
| B1 | Have you ever enrolled a first aid course or received first aid training? (Yes/No) | (Carruth et al., 2010; Chen et al., 2020; Dixe & Gomes, 2015; Jamaludin et al., n.d.; Khatatbeh, 2016; Mobarak et al., 2015; Wei et al., 2013) |
| B2 | Have you obtained the certification after completing the first aid training? (Yes/No) |  |
| B3 | Where have you attended first aid courses? (Yes/No) | (Kuramoto et al., 2008; Mobarak et al., 2015) |
| B4 | When was the last time (which year) you received first aid training? | (Chair et al., 2014; Dixe & Gomes, 2015) |
| B5 | Why do you want to take a first-aid training course? (multiple choices) | (Chair et al., 2014; Khatatbeh, n.d.; Kuramoto et al., 2008) |
|  | 1. Interesting in the first aids |  |
|  | 2. Encouragement of parents |  |
|  | 3. I want to participate in the extra-curriculum activities |  |
|  | 4. I want to help relatives and friends |  |
|  | 5. My friends invited me |  |
|  | 6. It is a mandatory course in my school |  |
|  | 7. Others |  |
| B7 | What is the reason for not having attended first aid training courses? (multiple choices) | (Chair et al., 2014; Khatatbeh, n.d.; Kuramoto et al., 2008) |
|  | 1. Have no interest in first aid |  |
|  | 2. Have no time to participate in first aid training course. |  |
|  | 3. No information about the first-aid training course. |  |
|  | 4. It is unnecessary |  |
|  | 5. It is not a mandatory course in our school |  |
|  | 6. Other |  |
| B8 | Which sources of first-aid information do you usually refer to update? (multiple answers) | (Dixe & Gomes, 2015; Mobarak et al., 2015; Wei et al., 2013) |
|  | 1. Internet |  |
|  | 2. Facebook |  |
|  | 3. Relatives |  |
|  | 4. Friends |  |
|  | 5. Television |  |
|  | 6. Teachers |  |
|  | 7. Others |  |
| C | History of injuries | |
| C1 | During the past 12 months, how many times were you seriously injured that made participants to be off school for at least one day? (Yes/No) | (World Health Organization & Centers for Disease Control and Prevention (CDC), 2013) |
| C2 | During the past 12 months, what was the most serious injury that happened to you? | (World Health Organization & Centers for Disease Control and Prevention (CDC), 2013) |
|  | 1. I had a broken bone or a dislocation joint |  |
|  | 2. I had a cut or stab wound |  |
|  | 3. I had a head injury |  |
|  | 4. I had a bad burn |  |
|  | 5. Other |  |
| C3 | During the past 12 months, what was the major cause of the most serious injury that happened to you? | (World Health Organization & Centers for Disease Control and Prevention (CDC), 2013) |
|  | 1. I was in a motor vehicle accident or hit by a motor vehicle |  |
|  | 2. I fell |  |
|  | 3. Something fell on me |  |
|  | 4. I was in a fire or too near a flame or something hot |  |
|  | 5. I inhaled or swallowed something bad for me |  |
|  | 6. Others |  |
| D | Willingness and self-efficacy regarding first aid | |
|  | What is your level of willingness to perform first aid in these circumstances? (“fully disagree” to “fully agree”). |  |
| D1 | 1. If the victim is strangers, I will provide the first aid | (Chair et al., 2014; Ma et al., 2015; Parnell et al., 2006) |
|  | 2. If I were in a situation requiring first aid skills and I were the only person who can help, I would perform the first aid | (Engeland et al., 2002) |
|  | 3. If I were in situation requiring first aid skills and other people are present, I would perform the first aid | (Engeland et al., 2002) |
| D2 | What is your confidence level when you perform… | (Wei et al., 2013) |
|  | 1. Calling emergency |  |
|  | 2. Cardiopulmonary resuscitation |  |
|  | 3. Chest compression |  |
|  | 4. Mouth-to-mouth ventilation |  |
|  | 5. Immobilization of fracture |  |
|  | 6. Stopping bleeding |  |
| D3 | Which are factors that prevent you providing first aid? |  |
|  | 1. The most important factor | (Chew & Yazid, 2008; Kuramoto et al., 2008; Ma et al., 2015) |
|  | 2. The second important factor |  |
|  | 3. The third important factor |  |
|  | 4. Others |  |
| D4 | Which are factors that motivate you to provide first aid? | (Chew & Yazid, 2008; Engeland et al., 2002; Kuramoto et al., 2008; Parnell et al., 2006) |
|  | 1. Victim is my friend |  |
|  | 2. I am the only bystander at that time |  |
|  | 3. I have been trained the first aid skills |  |
|  | 4. The victim is my family |  |
|  | 5. Others |  |
| D5 | Do you want to participate in a first aid course? (Yes/No) | (Goniewicz et al., 2012; Joshua, 2006; Ma et al., 2015; Parnell et al., 2006) |
|  | Which are skills that you want to learn? | (Wei et al., 2013) |
|  | 1. Calling emergency |  |
|  | 2. Cardiopulmonary resuscitation |  |
|  | 3. Chest compression |  |
|  | 4. Mouth-to-mouth ventilation |  |
|  | 5. Immobilization of fracture |  |
|  | 6. Stopping bleeding |  |
|  | 7. Others |  |

**REFERENCE**

Carruth, A. K., Pryor, S., Cormier, C., Bateman, A., Matzke, B., & Gilmore, K. (2010). Evaluation of a school-based train-the-trainer intervention program to teach first aid and risk reduction among high school students. *The Journal of School Health*, *80*(9), 453–460. https://doi.org/10.1111/j.1746-1561.2010.00527.x

Chair, S., Hung, M. S., Lui, J. C., Lee, D. T., Shiu, I. Y., & Choi, K. (2014). Public knowledge and attitudes towards cardiopulmonary resuscitation in Hong Kong: A telephone survey. *Hong Kong Medical Journal*. https://doi.org/10.12809/hkmj134076

Chen, C.-M., Jyan, H.-W., Chien, S.-C., Jen, H.-H., Hsu, C.-Y., Lee, P.-C., Lee, C.-F., Yang, Y.-T., Chen, M.-Y., Chen, L.-S., Chen, H.-H., & Chan, C.-C. (2020). Containing COVID-19 Among 627,386 Persons in Contact With the Diamond Princess Cruise Ship Passengers Who Disembarked in Taiwan: Big Data Analytics. *Journal of Medical Internet Research*, *22*(5). https://doi.org/10.2196/19540

Chew, K. S., & Yazid, M. N. A. (2008). The willingness of final year medical and dental students to perform bystander cardiopulmonary resuscitation in an Asian community. *International Journal of Emergency Medicine*, *1*(4), 301–309. https://doi.org/10.1007/s12245-008-0070-y

Dixe, M. dos A. C. R., & Gomes, J. C. R. (2015). Knowledge of the Portuguese population on Basic Life Support and availability to attend training. *Revista Da Escola de Enfermagem Da USP*, *49*(4), 0640–0649. https://doi.org/10.1590/S0080-623420150000400015

Engeland, A., Røysamb, E., Smedslund, G., & Søgaard, A. J. (2002). Effects of first-aid training in junior high schools. *Injury Control and Safety Promotion*, *9*(2), 99–106. https://doi.org/10.1076/icsp.9.2.99.8702

Goniewicz, M., Chemperek, E., Nowicki, G., Wac-Górczyńska, M., Zielonka, K., & Goniewicz, K. (2012). First Aid education in the opinion of secondary school students. *Open Medicine*, *7*(6). https://doi.org/10.2478/s11536-012-0048-2

Jamaludin, T. S. S., Zakaria, M. A. B., Saidi, S., & Chan, C. M. (n.d.). *Knowledge, Awareness and Attitude of First Aid Among Health Sciences University Students*. 5.

Joshua, D. (2006). Joshua I.A (MPH) Otu A.A (MPH) Chukwumerije C.C (MBBS) Achie L.N (MSc) Makama J.G (FWCS, MPH) Zamani B.W (PhD). *Journal of Community and Health Sciences*, 7.

Khatatbeh. (n.d.). *First aid knowledge among University students in Jordan*. Retrieved December 26, 2019, from http://www.ijpvmjournal.net/article.asp?issn=2008-7802;year=2016;volume=7;issue=1;spage=24;epage=24;aulast=Khatatbeh

Khatatbeh, M. (2016). First aid knowledge among University students in Jordan. *International Journal of Preventive Medicine*, *7*(1), 24. https://doi.org/10.4103/2008-7802.174772

Kuramoto, N., Morimoto, T., Kubota, Y., Maeda, Y., Seki, S., Takada, K., & Hiraide, A. (2008). Public perception of and willingness to perform bystander CPR in Japan. *Resuscitation*, *79*(3), 475–481. https://doi.org/10.1016/j.resuscitation.2008.07.005

Ma, A., Wong, K., Tou, A., Vyas, L., & Wilks, J. (2015). CPR Knowledge and Attitudes among High School Students Aged 15-16 in Hong Kong. *Hong Kong Journal of Emergency Medicine*, *22*(1), 3–13. https://doi.org/10.1177/102490791502200101

Mobarak, A. S., Afifi, R. M., & Qulali, A. (2015). *First Aid Knowledge and Attitude of Secondary School Students in Saudi Arabia*. https://doi.org/10.4236/health.2015.710151

Parnell, M. M., Pearson, J., Galletly, D. C., & Larsen, P. D. (2006). Knowledge of and attitudes towards resuscitation in New Zealand high-school students. *Emergency Medicine Journal*, *23*(12), 899–902. https://doi.org/10.1136/emj.2006.041160

Wei, Y.-L., Chen, L.-L., Li, T.-C., Ma, W.-F., Peng, N.-H., & Huang, L.-C. (2013). Self-efficacy of first aid for home accidents among parents with 0- to 4-year-old children at a metropolitan community health center in Taiwan. *Accident Analysis & Prevention*, *52*, 182–187. https://doi.org/10.1016/j.aap.2012.12.002

World Health Organization & Centers for Disease Control and Prevention (CDC). (2013). *Global School-based Student Health Survey (GSHS)*. World Health Organization and Centers for Disease Prevention and Control. https://ncvc.dspacedirect.org/handle/20.500.11990/1425
