# Supplementary material for: The willingness to perform first aid among high school students and associated factors in Hue, Vietnam
Source: PLoS One. 2022 Jul 27;17(7):e0271567. doi: 10.1371/journal.pone.0271567 (PMC9328566; doi:10.1371/journal.pone.0271567)
Supplement: S4 Table — (DOCX) [file pone.0271567.s005.docx]

**S4 Table. Factor loading of items in the self-efficacy scale**

| **Items** | **Factor1** | **Factor2** | **Factor3** | **Uniqueness** |
| --- | --- | --- | --- | --- |
| Emergency call |  |  | 0.8917 | 0.195 |
| Cardiopulmonary resuscitation | 0.9857 |  |  | 0.135 |
| Chest compression | 0.9198 |  |  | 0.1417 |
| Mouth-to-mouth ventilation | 0.6095 |  |  | 0.3888 |
| Immobilization of fracture |  | 0.4828 |  | 0.4836 |
| Stopping bleeding |  | 0.8171 |  | 0.3675 |
