# Supplementary material for: The willingness to perform first aid among high school students and associated factors in Hue, Vietnam
Source: PLoS One. 2022 Jul 27;17(7):e0271567. doi: 10.1371/journal.pone.0271567 (PMC9328566; doi:10.1371/journal.pone.0271567)
Supplement: S5 Table — (DOCX) [file pone.0271567.s006.docx]

**S5 Table. The correlation matrix of the factors after the oblique rotation.**

| **Factors** | **Factor1** | **Factor2** | **Factor3** |
| --- | --- | --- | --- |
| Factor1 | 1 |  |  |
| Factor2 | 0.6809 | 1 |  |
| Factor3 | 0.04791 | 0.2162 | 1 |
