# Supplementary material for: The willingness to perform first aid among high school students and associated factors in Hue, Vietnam
Source: PLoS One. 2022 Jul 27;17(7):e0271567. doi: 10.1371/journal.pone.0271567 (PMC9328566; doi:10.1371/journal.pone.0271567)
Supplement: S6 Table — (DOCX) [file pone.0271567.s007.docx]

**S6 Table. Factor loading of items in the willingness scale**

| **Items** | **Factor1** | **Uniqueness** |
| --- | --- | --- |
| The victim being a stranger | 0.8145 | 0.3366 |
| Being the only person who can help | 0.6604 | 0.5639 |
| Performing the first aid when other people are present | 0.7028 | 0.5061 |
